# Investigation of the Mitochondrial *ATPase 6/8* and *tRNA^Lys^* Genes Mutations in Autism

**Published:** 2012-08-31

**Authors:** Fahimeh Piryaei, Massoud Houshmand, Omid Aryani, Sepideh Dadgar, Zahra-Soheila Soheili

**Affiliations:** 1. Department of Medical Genetics, National Institute of Genetic Engineering and Biotechnology (NIGEB), Tehran, Iran; 2. Special Medical Center, Tehran, Iran; 3. Department of Basic Biotechnology, National Institute of Genetic Engineering and Biotechnology (NIGEB), Tehran, Iran

**Keywords:** Autism, Mitochondria, Mutation, ATP ase6/8, *tRNA^Lys^*

## Abstract

**Objective:**

Autism results from developmental factors that affect many or all functional brain systems. Brain is one of tissues which are crucially in need of adenosine triphosphate (ATP). Autism is noticeably affected by mitochondrial dysfunction which impairs energy metabolism. Considering mutations within *ATPase 6*, *ATPase 8* and *tRNA^Lys^* genes, associated with different neural diseases, and the main role of *ATPase 6/8* in energy generation, we decided to investigate mutations on these mtDNA-encoded genes to reveal their roles in autism pathogenesis.

**Materials and Methods:**

In this experimental study, mutation analysis for the mentioned genes were performed in a cohort of 24 unrelated patients with idiopathic autism by employing amplicon sequencing of mtDNA fragments.

**Results:**

In this study, 12 patients (50%) showed point mutations that represent a significant correlation between autism and mtDNA variations. Most of the identified substitutions (55.55%) were observed on MT-ATP6, altering some conserved amino acids to other ones which could potentially affect *ATPase 6* function. Mutations causing amino acid replacement denote involvement of mtDNA genes, especially *ATPase 6* in autism pathogenesis.

**Conclusion:**

MtDNA mutations in relation with autism could be remarkable to realize an understandable mechanism of pathogenesis in order to achieve therapeutic solutions.

## Introduction

Autism as a neurodevelopmental disorder is diagnosed by three core-defining features: delayed social interaction, impaired verbal or nonverbal communication and restricted and repetitive behavior. This condition is categorized as a multifactorial disease. Regardless of a strong genetic basis for autism, its genetics is immensely complicated considering multigene interactions or rare mutations with major effects in autism spectrum disorders (ASD) (1). Developmental factors that affect many functional brain systems cause autism (2) and disturbance in the perfect timing of brain development (3).

Autism is noticeably suggested to be affected by mitochondrial dysfunction as a result of perturbations to mitochondrial structural proteins and tRNAs which are encoded by mtDNA. The concept which had posed that mtDNA dysfunction may contribute to develop autism led to follow-on investigations to find mutations which would be causative factors in autism. Oliveira et al. (4) explained observation of the criteria for respiratory chain disorders in 7% of school-age children with ASD. According to the study by Ramoz et al. (5) autism shows a strong association with single nucleotide polymorphisms within the *SLC25A12* gene. Moreover, Weissman et al. (6) has reported that the electron transport chain (ETC) complexes I and III deficiencies affect energy metabolism in patients with autism. According to Pons et al. (7) study, the A3243G mutation in *tRNA^Leu^* 1 results in mtDNA depletion. In the study by Graf et al. on a family with heterogeneous neurological disorders with G8363A in *tRNA^Lys^*, a boy showed the characteristics of autism, although no reports have confirmed the certainty of this mutation involvement in autism in that child (8). Concerning the involvement of tRNAs mutations as potential risk factors and the *ATPase 6/8* role in producing energy, the main objective of this study was to identify mutations of considerable importance in mitochondrial *ATPase 6/8* and *tRNA^Lys^* genes which indicate meaningful correlation with developing autism.

## Materials and Methods

### Subjects, samples and DNA extraction

In this experimental study, 58 unrelated cases in the 4.5-8 age range were randomly obtained who had been already ascertained with autism by the specialists. The diagnosis of autism was made by Diagnostic and Statistical Manual of Mental Disorders criteria (9), Autism Diagnostic Interview-Revised (10) and Autism Diagnostic Observation Schedule (11) checklists, respectively.

All the cases were checked for known secondary causes of autism- additional inclusion criteria- by physical examination (for detection of any dysmorphic feature or skeletal abnormality as exclusion criterion), neurological examination, brain MRI, Fragile X and Rett syndrome, molecular test, cytogenetic study, electroencephalography, visual evoked potentials and brainstem acoustic evoked response, metabolic screening test on dried blood sample by MS/MS and urine organic acids evaluation.

Eventually, 24 cases (17 males and 7 females) with inclusion criteria for primary autism were enrolled in the study after taking their informed consents to the genetic analysis. The peripheral blood samples were obtained and genomic DNA was extracted using a DNA extraction kit (Diatom DNA Extraction Kit, Genefanavaran, Tehran). This study was approved by the Board of Ethics of National Institute of Genetic Engineering and Biotechnology (NIGEB), Tehran, Iran.

### mtDNA amplification and sequencing

In order to screen the mutations of *MT-TK, MT-ATP6* and *MT-ATP8* genes, amplification was carried out using a set of primers as follows; ONPF25 and ONPR185; flanking *MT-NC7, MT-TK, MT-ATP6* and *MT-ATP8* as one fragment. Then, the PCR reactions were performed as the previous study (12). PCR products were separately electrophoresed through 1.5% agarose to insure an specific band. The nucleotide sequences of the amplicons belonged to 24 patients were determined by automated sequencing 3700 ABI machine using primer ONPF25 (Macrogene Seoul, Korea) in search of mutations or amino acid changes in *tRNA^Lys^* and *ATPase 6/8*. The obtained mtDNA sequences were aligned with a multiple sequences alignment interface CLUSTAL-X to compare with rCRS. Identified variations were confirmed by repeated analysis of both strands.

## Results

This study led us to identify 9 different point mutations in 12 patients (50%) out of our cases on *MT-ATP 6, MT-ATP 8, MT-NC7* and some parts of *MT-COII* genes which are shown in table 1. On the *ATPase 6/8* genes, the five observed mutations resulted in amino acid replacements. Most of these substitutions (55.55%) occurred on *MT-ATP6*. 22.22% of point mutations were found on *MT-COII* and 11.11% of point mutations were noted on each of *MT-NC7* and *ATPase 8*. Some of these subsitutions were similarly observed in different patients; albeit, more than one point mutation were detected in some patients. Based on sequencing result, all obsreved mutations were found to be homoplasmic. No mutations were identified on *tRNA^Lys^* in 24 patients.

**Table 1:** mtDNA point mutations identified in patients with autism


Nucleotide position	Locus	Amino acid change	Frequency (%)	Reported in other disease

G8251A	MT-CO2	G→G	2 (8.3)	(13)
G8269A	MT-CO2	(in stop codon)	1 (4.16)	(14)
A8271G	MT-NC7	(in noncoding region)	1 (4.16)	(15)
C8472T	MT-ATP8	P→L	3 (12.5)	(16)
C8684T	MT-ATP6	T→I	1 (4.16)	(17)
G8697A	MT-ATP6	M→I	5 (20.83)	(15)
A8701G	MT-ATP6	T→A	1 (4.16)	(18)
A8836G	MT-ATP6	M→V	3 (12.5)	(19)
G8865A	MT-ATP6	V→V	1 (4.16)	(20)


G; Glycine, P; Proline, L; Lysine, T; Threonine, I; Isoleucine, M; Methionine, A; Alanine and V; Valine.

## Discussion

In respiratory chain, a number of polypeptides which are encoded with cooperation between mtDNA and nucleus gather to form a delicate series of enzyme complexes by which ATP, the most vital source of energy, is generated. Complex V (ATP synthase), as the last enzyme which directly plays a part in producing ATP, includes 14 subunits of which ATPases 6 and 8 encoded by mtDNA. Any effective mutation in these subunits causes loss of ATP production in the tissues with the highest demand of energy, such as brain and muscle which would consequently get damaged and results in human disease. *ATPase 6* is known to be a fast-evolving gene (21); however, some disorders have been found in association with mutations on *ATPase 6*. Since some amino acid residues belonging to *ATPase 6* are conserved in human species, any changes in these residues are considered potentially pathogenic. In addition, some residues which have been sustained in other mammals and prokaryotes in common with humans are known as highly conserved. Therefore, replacing them with other residues can be undoubtedly pathogenic because of altering the tertiary structure of *ATPase 6* (22). Furthermore, mtDNA encodes 22 tRNA genes which are necessary for mtDNA-encoded polypeptides synthesis; hence, any mutation altering functional structures in the mtDNA coding region definitely would affect mitochondrial energy production.

In the present study, significant amount of mtDNA variations were observed as 12 patients (50%) showed different substitutions mutation. Approximately 55% of these mutations were identified in *ATPase 6* while 80% of them led to amino acid replacements. It is noted that G8697A were detected in 20.83% of our patients which led to change from methionine (Met) to isoleusine (Ile). Met is a conserved residue in its position. In addition, it, as a sulphur-containing amino acid, is usually found hidden within proteins and has a tendency to form β-sheets. Despite having a hydrophobic property, it is able to react with some electrophilic centers. On the contrary, Ile abounds in α-helixes and plays a role in ligand binding to proteins. So, structure and function of *ATPase 6* are very likely to undergo a change in such replacement. Substitution at position 8836, one of highly conserved nucleotides, was another point mutation which was identified to replace Met to valine (Val) in 12.5% of cases. Similar to Ile, Val, with a very small side chain, is found abundantly in α-helix structures. In the same patients, C8472T led into non-polar proline (Pro) to polar lysine (Lys) replacement. Pro is often observed at the end of helix, turns, or loops. Being situated Pro in a helix, the helix will have a slight bend due to the lack of the hydrogen bond. Also, Pro can exist in the cis-configuration in peptides in contrast to other amino acids which are found exclusively in the trans- form in polypeptides. So cis/trans isomerization can play an important role in the folding of proteins. In Lys, the amino group is greatly reactive and often participates in reactions at the active centers. These two amino acids are entirely different in their chemical properties. Thus, this replacement can cause inappropriate interaction between this position and other residues that ultimately could result in *ATPase 8* malfunction .There were not any mutations identified on *tRNA^Lys^* in 24 patients. As a result, substitution G8363A is not likely to be a potential risk factor in autism.

Indication of previous researches strengthen the hypothesis that deficiencies in each of mitochondrial functional components lead to defective energy metabolism in autism. On the other hand, mitochondria perform an essential role in the generation of reactive oxygen species (ROS) (23) which can result in genetic instability by oxidative stress and DNA damage (24). In this point of view, we analysed *ATPase 6/8* mutations which can potentially alter the efficiency of these genes’ product. Among identified mutations, only A8836G has been reported to be pathogenic in association with LHON-like optic neuropathies which this finding is compatible with our result. *ATPase 6/8* genes have been investigated in different neurodegenerative diseases, such as Huntington’s disease (HD) (25), Ataxia Telangiectasia (AT) (26), Friedreich’s Ataxia (FA) (12), Multiple Sclerosis (MS) (27) and Spino Cerebellar Ataxias (SCA) (28). G8697A has been identified in patients with AT, MS and SCA, causing a conserved amino acid replacement. Another substitution at 8684 has been observed in patients with HD, MS, FA and SCA. All these findings suggest that the mtDNA mutations might be involved in pathogenesis of mitochondrial dysfunction in neural disorders.

## Conclusion

Our data showed a relation between increased mtDNA variations and autism. However, pathogenesis of autism as a multifactorial disorder is much more complicated than it seems. We suggest follow-up population and haplogroup studies in order to achieve more information about whether these substitutions are polymorphisms or pathogenic mutations.
